# A bilateral four-headed brachialis muscle with a variant innervation: a cadaveric report with possible clinical implications

**DOI:** 10.1007/s00276-024-03315-y

**Published:** 2024-03-05

**Authors:** Maria Piagkou, George Triantafyllou, Aggelos Koutsougeras, Christos Koutserimpas, Dimitris Katsogiannis, Georgi Georgiev, Lukasz Olewnik, Nicol Zielinska, George Tsakotos

**Affiliations:** 1 Department of Anatomy, School of Medicine, Faculty of Health Sciences, National and Kapodistrian University of Athens, 75 Mikras Asias str., Goudi, 11527 Athens, Greece; 2 Department of Orthopaedics and Traumatology, “251” Hellenic Air Force General Hospital of Athens, Athens, Greece; 3 Department of Orthopaedics and Traumatology, University Hospital Queen Giovanna-ISUL, Medical University of Sofia, Sofia, Bulgaria; 4 Department of Anatomical Dissection and Donation, Medical University of Lodz, Lodz, Poland

**Keywords:** Brachialis muscle, Median nerve, Musculocutaneous nerve, Radial nerve, Variation, Accessory heads

## Abstract

**Purpose:**

Anterior compartment muscles of the arm present high morphological variability, with possible clinical significance. The current cadaveric report aims to describe a bilateral four-headed brachialis muscle (BM) with aberrant innervation. Emphasis on the embryological background and possible clinical significance are also provided.

**Methods:**

Classical upper limb dissection was performed on an 84-year-old donated male cadaver. The cadaver was donated to the Anatomy Department of the National and Kapodistrian University of Athens.

**Results:**

On the left upper limb, the four-headed BM was supplied by the musculocutaneous and the median nerves after their interconnection.

On the right upper limb, the four-headed BM received its innervation from the median nerve due to the musculocutaneous nerve absence.

A bilateral muscular tunnel for the radial nerve passage was identified, between the BM accessory heads and the brachioradialis muscle.

**Conclusion:**

BM has clinical significance, due to its proximity to important neurovascular structures and frequent surgeries at the humerus. Hence, knowledge of these variants should keep orthopedic surgeons alert when intervening in this area. Further dissection studies with a standardized protocol are needed to elucidate the prevalence of BM aberrations and concomitant variants.

## Introduction

The anterior arm compartment is composed of the biceps brachii (BB), the coracobrachialis (CB), and the brachialis muscle (BM). Typically, the BM originates from the ventral surface of the distal half of the humerus and inserts into the coronoid process and the ulnar tuberosity. The BM is located beneath the BB and contributes to the cubital fossa formation. The BM acts as the primary flexor of the forearm [1]. According to classical anatomy textbooks, the BM typically is a one-headed muscle, while some cadaveric dissections evidence has disputed this theory [2]. The BM seems to be divided into two (superficial and deep) heads [1]. Typically, the innervation of the anterior arm compartment muscles derives from the musculocutaneous nerve (MCN) [1]. However, multiple studies have demonstrated the BM dual innervation, by the MCN and the radial nerve (RN) [1]. Small branches from the RN or the median nerve (MN) may be discovered during meticulous dissections. BM morphology and innervation present surgical interest, as it has been used for annular ligament [3], and for finger and wrist extensor tendons’ reconstruction [4]. The BM has an impact on the elbow joint stability [5].

The current cadaveric report describes a bilateral four-headed BM variant with an atypical innervation and discusses possible clinical implications. The embryological background is also emphasized.

## Case description

The dissection of the 84-year-old donated male cadaver was performed at the Dissection Hall of the Anatomy Department of the School of Medicine of the National and Kapodistrian University of Athens. The donation was completed through the “Body Donation Program” after written informed consent. Skin, subcutaneous fat, and superficial fascia of the upper limb were dissected, and all muscles of the anterior arm compartment were exposed from their proximal to their distal attachment. The lateral, medial, and posterior cords of the brachial plexus were visualized, and the muscles were carefully examined for a typical or variant attachment, morphology, and innervation. Upper limbs were free of any physical deformity or trauma. An electronic digital caliper was used for all measurements (*Mitutoyo Corporation, Kawasaki-shi, Kanagawa, Japan*), and each measurement was performed twice with an accuracy of up to 0.1 mm.

**Fig. 1:**
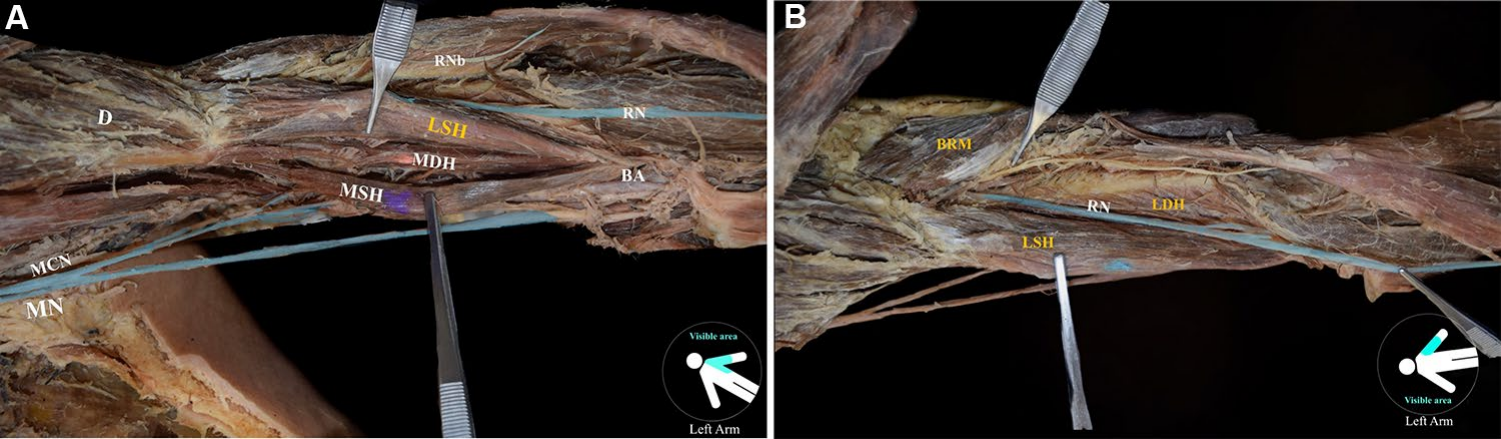
**A** The variant left-sided brachialis muscle (four-headed) coexisted with an atypical neural supply by the musculocutaneous nerve (MCN) and the median nerve (MN) after their interconnection. The main superficial, and deep heads (MSH and MDH), and the lateral superficial head (LSH), D: deltoid muscle; RNb: radial nerve branch; BA: brachial artery. **B** The left-sided variant brachialis muscle with the lateral superficial and deep heads (LSH and LDH) and the radial nerve (RN) coursing into a muscular tunnel between the lateral heads and the brachioradialis muscle (BRM). D: deltoid muscle; RNb: radial nerve branch; BA: brachial artery

**Fig. 2:**
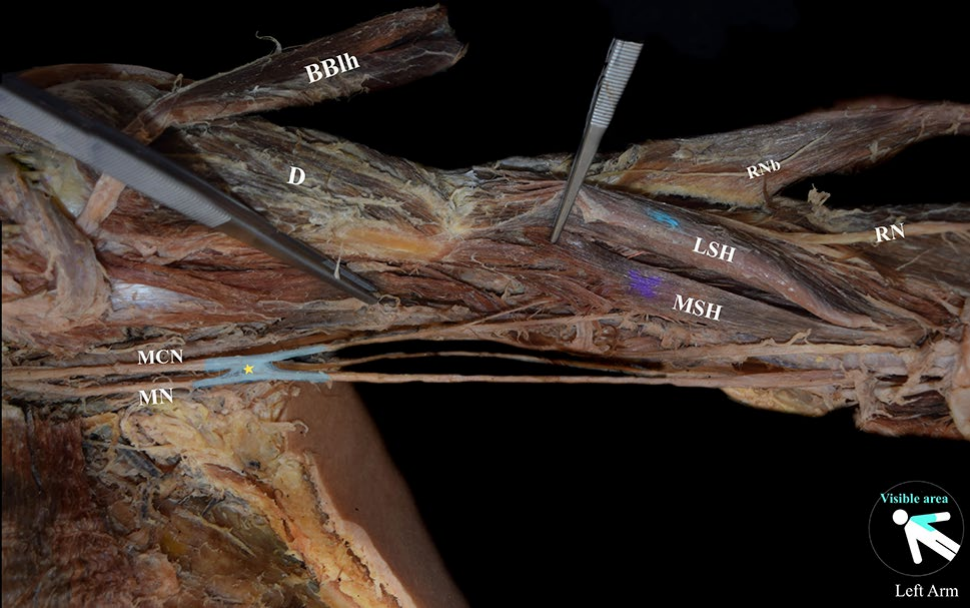
The complex innervation of the anterior compartment arm muscles and the musculocutaneous (MCN) and median (MN) nerve interconnection (*). BBlh: biceps brachii long head; D: deltoid muscle; RNb: radial nerve branch; MSH: main superficial brachialis head; LSH: lateral superficial brachialis head; RN: radial nerve

*On the left arm,* BM is constituted of four heads, two main (main superficial and deep heads—MSH and MDH) and two lateral (lateral superficial and deep heads—LSH and LDH) heads. The MSH and MDH originated from the deltoid tuberosity (proximal anteromedial surface) (Fig. 1A). The LDH originated from the anterior aspect of the distal half of the humerus, with borders distally to the deltoid tuberosity (anterolateral surface) and the lateral supracondylar ridge (Fig. 1B). The LSH, at its origin, was fused with the deltoid muscle (Fig. 1A). All heads were inserted in common into the ulnar tuberosity. An interconnection of the MCN with the MN was identified, and both nerves provided innervation to the variant BM (Fig. 2). The RN was identified into a muscular tunnel, formatted between the BM’s two lateral heads and the brachioradialis muscle (BRM) (Fig. 1B). RN branches to the BM were not identified.

**Fig. 3:**
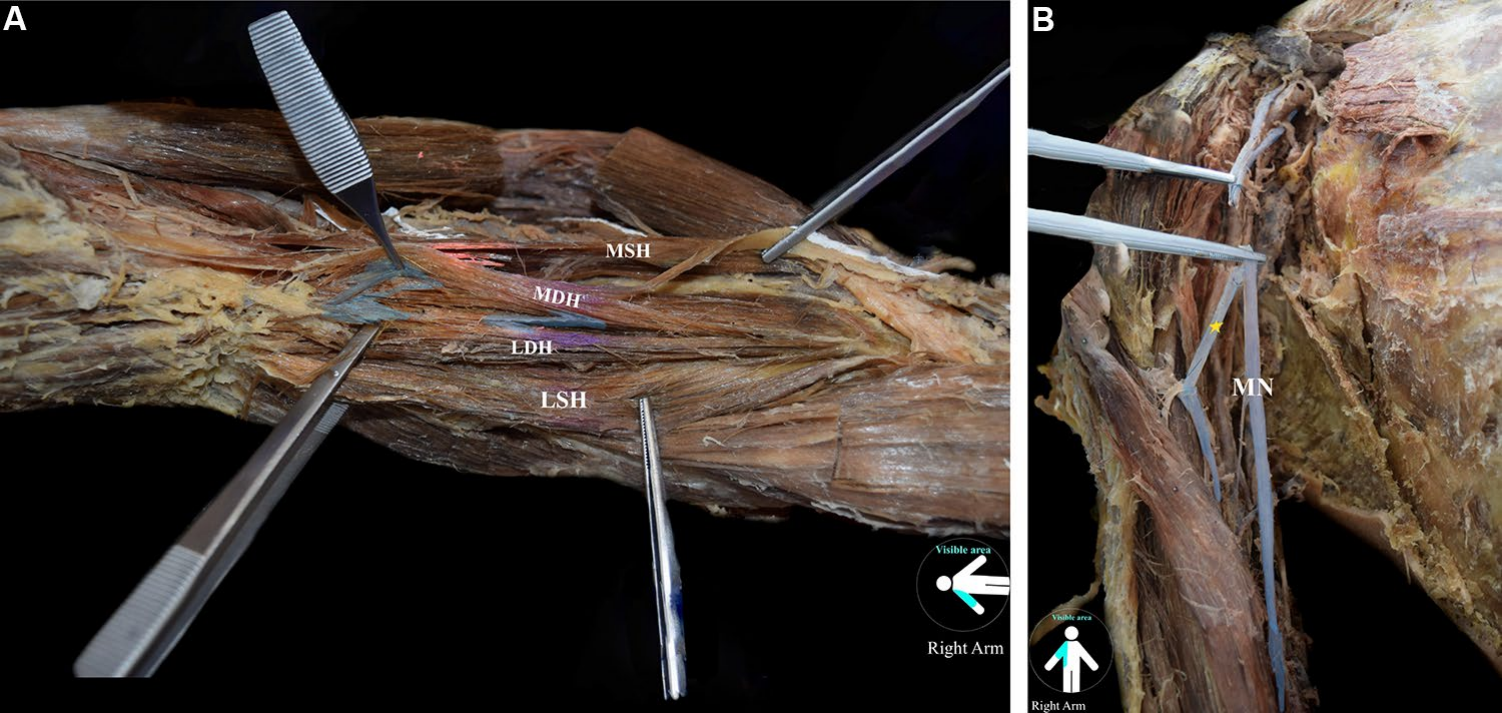
**A** The right-sided variant brachialis muscle coexisted with an absent musculocutaneous nerve (MCN). The main superficial and deep heads (MSH and MDH) and the lateral superficial and deep heads (LSH and LDH) of the four-headed brachialis muscle are depicted. **B** The right-sided variant brachialis muscle coexisted with an absent musculocutaneous nerve (MCN). The median nerve (MN) provides innervation to the anterior compartment muscles by a muscular branch (*)

*On the right arm,* the BM was also four-headed, with two main (MSH and MDH) and two lateral (LSH and LDH) heads (Fig. 3A). The MSH and MDH originated from the proximal humeral third (anteromedial surface), and the LSH at its origin was fused with the DM. The LDH originated from the anterior aspect of the distal humeral half, with borders distally to the deltoid tuberosity (anterolateral surface) and the lateral supracondylar ridge. The LDH and SDH partially fused at the BM upper third. All heads formed a common tendon at the cubital fossa and were inserted into the ulnar tuberosity. The RN coursed into a muscular tunnel formed by the LDH and the BRM, while the RN supply to the BM was not identified. The MCN was absent, and the anterior arm compartment muscles received their innervation from the MN (Fig. 3B). The BM morphometric measurements are summarized in Table 1.

**Table 1:** Morphometric parameters of the bilaterally variant brachialis muscle (BM). Length is expressed in mm

Parameters	Main heads		Lateral heads	
Lengths by side (in mm)	Superficial head	Deep head	Superficial head	Deep head
Left side	148.2	166.5	177.1	150.8
Left-side common tendon	13.5			
Right side	166.4	188.6	164.3	156.5
Right-side common tendon	18.2			

## Discussion

### Brachialis muscle (BM) embryological development and possible variants

The upper limb muscles develop from a proliferation of the mesoderm of the somatopleure in the lateral region of the body, at the level of the lower six cervical and upper two thoracic segments [1, 6]. Especially for the upper extremity musculature, all the upper limb muscle heads have a common embryological origin from the muscle primordia, which develops approximately on the 28th developmental day, from dorsolateral somite cells that migrate into limb buds [1]. The common pre-muscular mass develops in the 11 mm length embryo, and the three anterior compartment muscles have been differentiated in a 14–16 mm length embryo [1]. The complex developmental procedure follows a dorsoventral and a proximal to distal direction, under the control of multiple factors and signaling pathways, such as sonic hedgehog protein and Wnt signaling [1].

Developmental alterations can lead to variant muscles, with additional heads [1]. Some primitive muscle cells undergo apoptosis, while a failure during this procedure is responsible for the development of muscle variants [6], which may exert an influence on vascular and nerve development; that’s why neural alterations are frequently identified as coexisting with muscle variants [1].

### Morphological variability of brachialis muscle (BM)

In the current case, a bilateral four-headed BM was identified. Recently, BM morphological variability has gained interest as few cadaveric studies and reports have been investigating its anatomy and morphology [2, 6–10], assessing the variability in the number and morphology of its heads, their origin, insertion, and innervation. The BM morphology has been identified as highly variable in the old anatomical manuscripts [8, 10]. Macalister [8] described 27 morphological variants of the muscle. BM can be divided into two or more parts; fibers can be seen originating from the BM into the BRM or the fascia over the MN and brachial artery [8]. Testut [10] identified the muscle division into three, or four heads with different insertions. He reported that the muscle could be fused with the DM, the CB, and the BB [10]. Testut [10] referred to the BM comparative anatomy, highlighting that the two-headed muscle is considered the typical anatomy in rodents, rabbits, crocodiles, and black bears. Mori [9] recorded the BM division into two heads in 24% of his sample. Ilayperuma et al. [2] in their dissections on 240 upper limbs, identified a two-headed BM in all cases. The BM superficial head was larger and was located anteriorly and proximally to the deep head. The superficial head was inserted into the ulnar tuberosity, and the deep head into the ulna coronoid process [2]. Contrariwise, in the current case, all BM heads formed a common tendon and were inserted into the ulnar tuberosity. Loukas et al. [7] described an accessory BM that crossed the brachial artery and the MN, and Kaliappan et al. [6] identified an accessory BM that descended distally to the forearm, fused with the pronator teres, and crossed the cubital fossa neurovascular bundle.

Thus, BM morphology presents quite a few variants; the presence of supernumerary heads [1] and accessory muscle bundles [6, 7]. Nevertheless, its innervation raises a lot of interest. In the current case, the BM was supplied by the MN (right side) and by both MN and MCN (left side). The MCN absence in our case, justified the MN innervation of the anterior compartment muscles. To visualize, extra thin and particularly intramuscular innervation from the RN, special care should be given in cadavers’ dissection protocol by using magnified glasses. One of the most adequate staining for this visualization is Sihler’s staining, according to Won and co-authors [11], who identified a BM complex innervation from the MCN, RN, and MN in various combinations. According to their classification, BM type I innervation was provided only by the MCN (25%), type II was both by the MCN and RN (55%), type III by the MCN, and MN (15%) and type IV innervation was provided by all three nerves (5%). Ilayperuma et al. [2] in 83.3% of their sample, observed the RN contribution to the BM deep head along with the MCN. Guerri-Guttenberg and Ingolotti [12] classified MCN variants and identified the MCN absence in 3.6% of their sample. They further subclassified the MCN absence into two types. In type 1, the MN innervated the anterior arm compartment muscles from a common trunk (1.8%) [12].

### Clinical implications of the brachialis muscle variants

The BM is commonly divided during certain surgical approaches to the humerus, especially in cases of supracondylar fractures in children. Due to its significance and proximity to crucial neurovascular structures, extreme caution is necessary for this approach [13]. Splitting the BM anteriorly or anterolaterally carries a considerable risk of injuring the nerve branches, even when the muscle may receive a dual innervation by the MCN and RN [14]. In the current case, the RN was identified in a muscular tunnel, formatted between BM accessory lateral heads and BRM. This tunnel could be a potential site of compression for the RN, the so-called “high RN entrapment” due to its location above the cubital fossa [15]. BM multiple heads, such as in the current case may complicate the surgical approach; hence, knowledge of these variants is important to avoid iatrogenic intraoperative injury and adverse effects. Some surgeons have explored a lateral approach that avoids splitting the BM, although concerns persist about the potential rise in post-operative nerve complications (palsies) due to the required manipulation and dissection [16]. BM tendon transfer represents a viable technique for the flexors digitorum profundus and the flexor pollicis reconstruction; this muscle could be also transferred to the pronator teres, the extensors carpi radialis brevis, and longus to restore wrist extension and/or pronation. Given that forearm muscles may not always be accessible for such procedures, the BM serves as an alternative donor in these applications [4]. BM variant innervation could impede the transfer of the BM branch of the MCN to the anterior interosseus nerve. Combined with a lateral antebrachial cutaneous nerve graft this technique could be used to reconstruct thumb and finger flexion [14].

### Limitations

The current report has some limitations. The cadaver was used for educational purposes and was initially dissected by students and then by the senior authors. The dissection was not performed with magnifying loops or an operating microscope, and neither a Sihler’s stain was available. Thus, the visualization of RN small branches and the intramuscular innervation of BM was not possible. Also, the vascularization of the atypical muscle was not possible to be identified.

## Conclusion

The current cadaveric report presents a bilateral four-headed BM, with a variant innervation. Anterior compartment muscles of the arm, including the BM, depict high morphological variability with concomitant neural alterations. The knowledge of those variations is important for clinicians and interventionists in the area, to diagnose pathologies or prevent iatrogenic lesions, intraoperatively. Further dissection studies with a standardized protocol are needed to elucidate the prevalence of the BM aberrations, and concomitant variants.

## Data Availability

No datasets were generated or analysed during the current study.
